# Cardio-metabolic health and sleep quality in adults at risk for Type 2 Diabetes using the Fos Biomedical Non-Transdermal Patch System via photo-biomodulation: A randomized, placebo-controlled crossover trial

**DOI:** 10.1016/j.conctc.2025.101448

**Published:** 2025-02-15

**Authors:** Valentine Y. Njike, Rockiy G. Ayettey, Judith A. Treu, Beth Patton Comerford, Maureen Onuigbo

**Affiliations:** a Yale-Griffin Prevention Research Center, USA; b Yale-Griffin Prevention Research Center, 130 Division Street, Derby, CT 06418, USA; c Griffin Hospital, 130 Division Street, Derby, CT 06418, USA

**Keywords:** Pre-diabetes, Metabolic syndrome, Type 2 diabetes, Phototherapy, Photo-biomodulation, Fos Biomedical patch, Metabolic control, Sleep quality

## Abstract

**Background:**

The impact of the Fos Biomedical non-transdermal patch system (NTPS) that stimulates the skin with low light levels to generate photo-biomodulation (PBM) effects on cardio-metabolic health and sleep quality is unclear. We examined the impact of FBPS compared with placebo on cardio-metabolic risk and sleep quality in persons at risk for type 2 diabetes mellitus (T2DM).

**Methods:**

The study was a randomized, controlled, double-blind, crossover trial of 39 adults (mean age 64.4 years; 28 women, 11 men; 38 Caucasians, 1 African American) at risk for T2DM assigned to one of two possible sequence permutations of two treatments (Fos Biomedical NTPS and placebo), with an 8-week washout period. Fos Biomedical NTPSs are designed to stimulate the skin with low light levels to produce PBM effects. Participants were instructed to apply the active or placebo patches above and below the belly button for 12 h each day for 12 weeks. Primary outcome measure was glycated hemoglobin (HbA1c). Secondary outcome measures included insulin sensitivity, lipid profile, blood pressure, body composition, C-reactive protein, endothelial function, and sleep quality.

**Results:**

Compared with the placebo, the Fos Biomedical NTPS did not improve glycemic control: HbA1c (0.1 ± 0.2 % vs. 0.1 ± 0.2 %; p = 0.5154). Compared with placebo, Fos Biomedical NTPS reduced endothelial function (−1.7 ± 12.1 % vs. 3.9 ± 10.0 %; p = 0.0344) while other markers of cardiovascular risk (i.e., body composition, blood pressure, lipid profile, and inflammatory biomarker) and sleep quality were unaffected (p > 0.05).

**Conclusions:**

Photo-biomodulation generated from Fos Biomedical NTPS did not improve biomarkers of cardio-metabolic risk and sleep quality among those at risk for T2DM.

**Clinical trial registration number:**

NCT05628597.

## Abbreviations

NTPS: non-transdermal patch system
PBM: photo-biomodulation
T2DM: type 2 diabetes mellitus
HbA1c: glycated hemoglobin
CVD: cardiovascular disease
CCO: cytochrome *c* oxidase
NO: nitric oxide
IRB: Institutional Review Board
BMI: Body Mass Index
ATP: Adult Treatment Panel
PRC: Prevention Research Center
TG: triglycerides
PI: Principal Investigator
GRAS: Generally Recognized as Safe
FDA: Food and Drug Administration
HPLC: High Performance Liquid Chromatography
ADA: American Diabetes Association
HOMA-IR: Homeostatic Model Assessment of Insulin Resistance
BP: Blood Pressure
CRP: C-reactive Protein
hs-CRP: high sensitivity CRP
Tchol: total cholesterol
HDL: high-density lipoprotein
LDL: low-density lipoprotein
EF: Endothelial Function
BARS: brachial artery reactivity
FMD: flow-mediated dilatation
PSQI: Pittsburgh Sleep Quality Index
ASA24: Automated Self-Administered 24-Hour Recall
PAR: Physical Activity Recall

## 1 Background

People with pre-diabetes (higher than normal blood sugar levels, but not high enough to be diagnosed as type 2 diabetes) and/or metabolic syndrome are at increased risk for developing type 2 diabetes mellitus (T2DM), cardiovascular disease (CVD), stroke, and a higher risk of mortality [1,2]. The combination of pre-diabetes and metabolic syndrome is associated with an even higher (21-fold) risk for T2DM when compared with healthy individuals [1]. Insulin resistance and excess body weight, particularly due to excess central body fat are common in both pre-diabetes and metabolic syndrome [3]. An inconsistent sleep schedule or a general lack of sleep has been associated with pre-diabetes and metabolic syndrome [[4], [5], [6], [7], [8], [9]]. Sleep disturbance is associated with poor cardio-metabolic control (hypertension, dyslipidemia, and reduced insulin levels after eating) [5,10,11]. Therefore, improving sleep patterns is likely to improve cardio-metabolic risk factors among those at risk for T2DM.

Lifestyle practices that promote good sleep hygiene and reduce stress have been associated with a lower risk of T2DM and the control of cardio-metabolic risk factors among those at risk for T2DM [12] In addition, consistent sleep patterns have also been associated with improved glycemic control in T2DM [13]. Phototherapy is thought to help improve sleep patterns and length of sleep in persons with circadian rhythm sleep disorders [14]. Furthermore, in a meta-analysis by Wang et al. [15] phototherapy therapy was shown to improve symptoms of vascular complications and quality of life that are linked to diabetes [16]. In an animal model, phototherapy has been shown to reduce abdominal fat [17]. In addition, phototherapy has also been associated with improved insulin sensitivity in T2DM [16,18].

Phototherapy, also known as photo-biomodulation (PBM) or low-level light therapy, has been known by scientists for almost 50 years but still has not gained widespread acceptance, largely due to uncertainty about the mechanisms of action. In recent years, much knowledge has been gained in this area [19]. The primary site of light absorption in mammalian cells has been identified as the mitochondria, and more specifically, cytochrome *c* oxidase (CCO) [20], an enzyme that contains both heme and copper centers [19] and is known to reduce oxygen to water at the end of the mitochondrial respiratory chain [21]. CCO has recently been shown to have an additional enzymatic activity: the reduction of nitrite to nitric oxide (NO) upon exposure to low-intensity light [21]. The absorption peaks of CCO are in the visible (420–450 nm and 600–700 nm) and the near-infrared (760–980 nm) spectral regions.

The effects of the Fos Biomedical non-transdermal patch system (NTPS), which utilizes the concept of phototherapy on cardio-metabolic risk factors and sleep quality in persons at risk for T2DM are unclear. This randomized crossover placebo-controlled trial assessed the impact of the Fos Biomedical NTPS use on cardiometabolic risk factors and sleep quality among adults at risk for T2DM. Specifically, we hypothesized that the daily use of the Fos Biomedical NTPS for 12 weeks, compared with the placebo patch system, would improve cardio-metabolic risk factors markers and sleep quality in adults at risk for T2DM.

## 2 Methods

### 2.1 Study design

This was a randomized, double-blind, placebo-controlled, crossover trial designed with two treatment assignments (i.e., Fos Biomedical NTPS and placebo patch) to compare the effects of the daily use for 12 weeks of each treatment assignment on cardio-metabolic risk factors and self-reported sleep quality in adults at risk for T2DM. Enrolled participants were randomized to one of two possible sequence permutations and they underwent repeated measures before and following daily use of the Fos Biomedical NTPS or placebo patch system for 12 weeks, with an 8-week washout period between treatment assignments. This study was conducted in a community hospital (Griffin Hospital) in Derby, Connecticut in the United States. The study was approved (#2021–10) by the Griffin Hospital Institutional Review Board (IRB) and was registered at the ClinicalTrials.gov website (NCT05628597).

### 2.2 Study participants

Participants were recruited through flyers, social media, and newspaper advertisements in the Lower Naugatuck Valley in Southern Connecticut. The study coordinator pre-screened potential participants for eligibility with a structured telephone interview utilizing predefined inclusion and exclusion criteria depicted in the study protocol. Inclusion criteria included males >40 years of age; post-menopausal females not currently on hormone replacement therapy; non-smokers; overweight with Body Mass Index (BMI) ≥25 kg/m^2^; and at risk for T2DM (i.e., either meeting the Adult Treatment Panel (ATP) III criteria for metabolic syndrome [22] or pre-diabetes with HbA1c in the range of 5.7–6.4 %). Exclusion criteria included failure to meet inclusion criteria; anticipated inability to complete study protocol for any reason; Type 1 or T2DM; personal history or family history of skin cancer; having lupus; having liver disease; use of lipid-lowering or antihypertensive medications, unless stable on medication for at least 3 months and willing to refrain from taking medication for 12 h prior to clinical outcome measures assessment; regular use of high doses of vitamin E or C; use of insulin, glucose-sensitizing medication, vasoactive medication (including glucocorticoids, antineoplastic agents, psychoactive agents, or bronchodilators) or nutraceuticals; regular use of fiber supplements; sleep apnea; coagulopathy, known bleeding diathesis, or history of clinically significant hemorrhage; or current use of warfarin; and known allergic or dermatological reactions to any of the components of the patch system or placebo - polyethylene, silicone, or acrylate adhesive – that could have contact with the skin of study participants during their use of the product.

Those who met preliminary eligibility criteria and agreed to participate were invited to undergo clinical eligibility screening and were asked to sign a written consent form approved by the Griffin Hospital IRB. All participants were informed of the option of discontinuing participation at any time during the study. The clinical screening physical examination consisted of weight, height, waist circumference, and blood pressure measures obtained by a clinical research specialist using calibrated equipment in the Prevention Research Center (PRC) vascular laboratory. In addition, participants underwent a fasting blood profile for lipids (total cholesterol, HDL, LDL, and triglycerides (TG), fasting glucose, and glycated hemoglobin (HbA1c) serum assessment at the Griffin Hospital laboratory. The findings of the physical examination and the blood test were used to establish whether a potential candidate met the criteria of being at risk for T2DM (i.e., having metabolic syndrome and/or pre-diabetes). Thirty-nine participants who were at risk for T2DM and who met the eligibility criteria were enrolled in the study.

### 2.3 Randomization and masking

Participants enrolled in the study were randomized to 1 of 2 sequence permutations of the Fos Biomedical NTPS and a placebo patch system by the Principal Investigator (PI) using SAS software for Windows version 9.4 (SAS Institute, Cary, NC). Each sequence permutation consisted of a 12-week treatment phase, followed by an 8-week washout phase, followed by an alternate 12-week treatment phase. The study coordinator assigned the participants to 1 of the 2 sequence permutations generated with SAS by the PI. The investigators, project coordinator, clinical research specialist (assessing the outcome measures), and study participants were masked to treatment assignments throughout the study. Both of the two patch systems (Fos Biomedical NTPS and placebo) were provided to us by the study funder (Fos Biomedical). The two patch systems had a similar appearance and texture. The products were color-coded as “blue” and “pink” by Fos Biomedical. The description of the products was sent by Fos Biomedical in a sealed envelope to the PRC before the initiation of the study, that was kept in a locked cabinet throughout the study. After the statistical analyses, the envelope was opened to reveal the descriptors of the treatment assignments.

### 2.4 Procedure

*Fos Biomedical Non-transdermal Patch System*: The test products to be evaluated were provided to the study team by Fos Biomedical, a Florida-based company, that researches and develops products to improve human health. The products use a patented technology designed to elicit specific biochemical responses from the body in response to wavelengths of light. The light is delivered via a non-transdermal patch embedded with novel crystal materials that, when activated by body heat, reflect low levels of light in the infrared and visible band to stimulate the surface of the skin. The specific product (Panabetic) used in the study intervention was developed to help manage blood glucose levels in persons with pre-diabetes. **This** single-use and non-sterile product uses a combination of 2 patches known as the patch system. Each patch is made of a non-woven material contained between 2 layers of clear water-resistant polyethylene. The top layer of water-resistant polyethylene laminate film is sealed to the bottom layer. The bottom layer is a medical-grade polyethylene tape coated with a hypoallergenic pressure-sensitive acrylate adhesive used to adhere the patch to the body. Between the 2 layers is a non-woven material treated with water-based solutions containing distilled water, glycerol, and potassium sorbate. The compounds are GRAS-listed (Generally Recognized as Safe) by the Food and Drug Administration (FDA). The patches are designed to reflect very specific wavelengths of light, i.e., in the range of 600–1000 nm within the red and near-infrared wavelengths. When placed on the skin surface, the patches can safely stimulate the skin with low levels of light, thus producing PBM effects. Each set of patches (patch system) is designed for single use within a period of up to 12 h.

*Placebo Patch System*: The placebo patch system was packaged in an identical fashion to the active patch system, except for being packaged in a different color and had the same instructions for use. The patches inside the package appeared visually identical to those of the Fos Biomedical NTPS. The placebo patches contained saline only and did not function as reflectors in a similar fashion to the Fos Biomedical NTPS.

*Application of the Patch Systems:* For each of the 12-week treatment phases, the study participants were provided with enough single-use patch systems (i.e., a total of 84 cartons of single-use patch systems) to cover the length of the treatment phase. Each single-use patch system was packaged in a carton that includes 2 patches, along with instructions for use. Each patch had either the name P1 or P2 printed on the back. Study participants were instructed to remove the plastic backing on each patch, place a P1 patch above the belly button, and place a P2 patch below the belly button. To standardize the distance from the belly button, they were instructed to use a distance of two fingers above (P1) or below (P2) the belly button. To standardize the time of day for wearing the patches, while maximizing the likelihood of compliance, we asked each participant to wear the patches for a 12-h period during waking hours in accordance with their current routines, during a time when the patches would not get wet from showering or bathing. For example, someone who finished taking a shower at 8:00 a.m. could apply the patches before getting dressed and remove the patches at around 8:00 p.m. or before going to sleep.

*Compliance:* During the study assessment visit at the end of each treatment assignment phase, the study coordinator met with participants to assess compliance with the treatment assignment. Compliance was assessed by self-report and by collecting the returned product using a log at the end of each 12-week treatment assignment. For each of the two treatment assignment phases, good compliance was defined as >80 % use of treatment during the respective 12-week treatment assignment.

*Maintaining Dietary Pattern and Physical Activity Level:* To control for the potential confounder of any dietary and physical activity changes made during the study on cardio-metabolic risk factors, participants were asked to maintain their usual dietary and physical activity patterns, i.e., eating and activity habits, during their time of involvement in this study.

## 3 Study outcome measures

Participants were asked to fast at least 8 h before the assessments of outcome measures. The outcome measurements were evaluated before and after the two 12-week treatment assignments (i.e., the Fos Biomedical NTPS or placebo).

### 3.1 Primary outcome measure

*Glycated Hemoglobin (HbA1c)*: The G8 automated High-Performance Liquid Chromatography (HPLC) analyzer was used to quantify HbA1c from the blood samples of the study participants at each visit. HbA1c variant analysis mode used non-porous ion-exchange HPLC for rapid, accurate, and precise separation of the stable form of HbA1c from other hemoglobin fractions. Analysis was conducted without offline specimen pretreatment or interference from the Schiff base. The analyzer dilutes the whole blood specimen with Hemolysis and Walsh solution, then injects a small volume of this specimen onto the TSKgel G8 SHi variant column. Separation was achieved by using the difference in ionic interaction between the cations exchange group on the column resin surface, and the hemoglobin fractions were subsequently removed from the column by performing a stepwise elution using the varied salt concentrations in the Elution Buffers SHi variants 1, 2, and 3. HbA1c levels were measured as the percentage of hemoglobin protein in the participants’ blood coated with sugar. HbA1c levels were used to gauge the average blood glucose level over a previous 2- to 3-month period, which is the lifespan of red blood cells. HbA1c levels predict future risk for T2DM [23]. According to the American Diabetes Association (ADA), HbA1c is considered a gold standard for monitoring glycemic control and is also used for screening and diagnosis of diabetes [24]. Higher values of HbA1c are associated with poor glycemic control and a greater risk of developing diabetes complications.

### 3.2 Secondary outcome measures

*Insulin Sensitivity*: Fasting glucose and insulin levels were measured at each visit. Homeostatic Model Assessment of Insulin Resistance (HOMA-IR) measures were assessed using the fasting glucose and insulin levels to gauge insulin sensitivity. HOMA-IR measures were calculated by the formula: fasting insulin (μU/mL) multiplied by fasting glucose (mg/dL) divided by 405 [25].

*Body Composition*: The clinical research specialist used Tanita SC-240 (Tokyo, Japan) to measure the participants’ calculated percentage of body fat%, total body water and visceral fat rating at each visit. The use of an impedance-based prediction equation for percent body fat, when compared with either densitometry or dual-energy X-ray absorptiometry - two current standards used to measure body composition - provided reliable estimates of percent body fat.

*Body Weight*: At each visit, the clinical research specialist measured the participants’ body weight in kilograms to the nearest 0.5 kg using a balance-type medical scale while participants were fasting and unclothed except for undergarments. BMI was calculated by dividing weight (in kilograms) by the square of height (in meters).

*Waist Circumference*: Participants' waist circumference was measured at each visit by the clinical research specialist using a standardized protocol. Participants were asked to stand, and a measurement tape was placed horizontally around each participant's middle, at the level of the belly button. The tape was kept snug around the participant's waist without compressing the skin, and waist circumference was recorded just after exhaling [26].

*Blood Pressure (BP)*: Systolic and diastolic BP were measured at each visit using a Dinamap Monitor Pro 100 (GE Healthcare, Piscataway, NJ) with the participant sitting in a quiet room for at least 5 min. Blood pressures were calculated as the mean value of 2 readings done 5 min apart for each participant.

*C-reactive Protein (CRP)*: CRP values were determined at each visit using a high-sensitivity CRP (hs-CRP) ELISA method. The VITROS chemistry products hs-CRP Reagent is a dual package containing ready-to-use liquid reagents. Samples, calibrators, and controls were mixed with reagent 1 containing a buffer. The addition of anti-CRP antibodies coupled to latex microparticles (reagent 2) produces an immunochemical reaction yielding CRP antigen-antibody complexes. The turbidity was measured spectrophotometrically at 660 nm. Once the calibration was performed for each reagent lot, the CRP concentration in each unknown sample was determined using the stored calibration curve and the measured absorbance obtained in the assay of the sample. The hs-CRP is more sensitive than the standard CRP test. The high hs-CRP level is associated with a greater risk of developing coronary artery disease, especially among persons with a 10–20 % chance of having a heart attack within 10 years.

*Serum Lipids*: At each visit, total cholesterol (Tchol), triglycerides (TG), and high-density lipoprotein (HDL) were obtained by direct measurements from the participants’ serum. Low-density-lipoprotein (LDL) were calculated using the formula: LDL = Tchol - (TG/5 + HDL) multiplied by HDL:Tchol ratio.

*Endothelial Function (EF)*: The brachial artery reactivity studies (BARS) methodology was employed as described in the published “Guidelines for Ultrasound Assessment of Endothelial-dependent Flow–mediated Vasodilation of the Brachial Artery.” [27] BARS assessments were conducted by a clinical research specialist before and at the end of each treatment phase. The measure of interest was flow-mediated dilatation (FMD) of the brachial artery. The FMD was measured as the percent change in brachial artery diameter from pre-cuff inflation to 60 to180-s post-cuff release.

*Pittsburgh Sleep Quality Index (PSQI)*: The PSQI questionnaire was administered to the study participants by the study coordinator before and at the end of each intervention phase. The PSQI is a self-rated questionnaire to assess perceived sleep quality and disturbances over a 1-month time interval. This 19-item instrument uses a Likert scale (ranging from 0 to 3) to assess seven clinically derived domains of sleep: sleep quality, sleep latency, sleep duration, habitual sleep efficiency, sleep disturbances, use of sleeping medication, and daytime dysfunction. The sum of scores for these seven components yields one global score. Clinical and clinimetric properties of the PSQI were assessed over 18 months with "good" versus "poor" sleepers. A global score >5 yielded a diagnostic sensitivity of 89.6 % and specificity of 86.5 % (kappa = 0.75, p < 0.001) in distinguishing good versus poor sleepers [28].

*Dietary Pattern*: Participants were asked at baseline and 12 weeks of each treatment phase to provide information on the foods and beverages consumed on 3 days of a week (i.e., 2 weekdays and 1 weekend day) to help track any variation in dietary patterns in the study. Participants completed 3 consecutive 24-h recalls using a web-based Automated Self-Administered 24-h Recall (ASA24) (available from the National Cancer Institute at http://riskfactor.cancer.gov/tools/instruments/asa24/), which guided them through the process of completing the recall data. Each participant received instructions to access the database using a unique study ID number and password. The data were reviewed by the study dietitian before statistical analyses.

*Physical Activity*: Physical activity was evaluated using the Seven-Day Physical Activity Recall (PAR) [29]. The PAR is one of the most widely used physical activity assessments in exercise science and epidemiological research. The popularity of this measure stems mainly from its versatility and relative ease of use for research applications. The PAR provides details regarding the duration, intensity, and volume (energy expenditure) of physical activity and can be used for different applications. Because it utilizes a one-week time frame, the data from the PAR are often considered representative of typical activity patterns.

*Assessment of Safety and Adverse Events:* Participants reported any adverse events experienced during the study to the study coordinator. Adverse events reported to the study coordinator were all presented to the PI, who informed the IRB.

### 3.3 Statistical analysis

The sample size estimations were based on the study's primary outcome measure, HbA1c. Using a standard deviation of 1 % in HbA1c, the sample size estimate provided at least 80 % power to detect a minimal improvement of 0.5 % in HbA1c between the Fos Biomedical NTPS and placebo, with a maximum allowable type I error of 5 %. Generalized linear models were used to compare the pre-post scores of the outcome measures between the Fos Biomedical NTPS versus placebo. Paired student t-tests were used to assess the difference from baseline to endpoints for each treatment assignment. Regression models were used to control for covariates (i.e., age, gender, race, calorie intake, physical activity level, compliance, and treatment sequence). All analyses at endpoints were based on the intention-to-treat principle to preserve randomization. SAS software for Windows version 9.4 (SAS Institute, Cary, NC) was used to conduct all statistical analyses. P-values <0.05 were considered statistically significant. Data are presented as mean ± standard deviation except otherwise stated.

## 4 Results

**Fig. 1:**
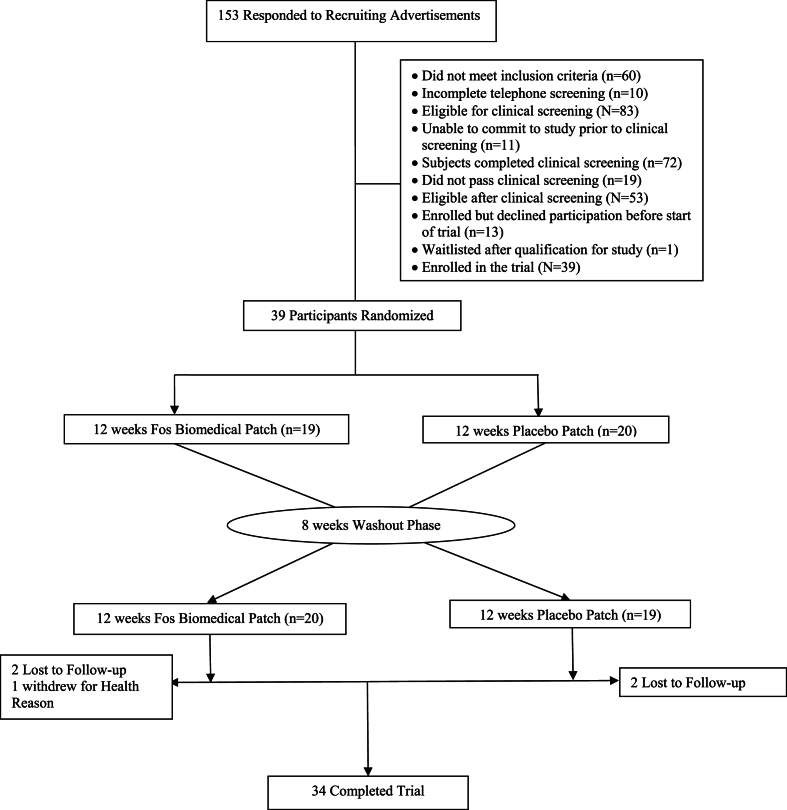
Flow of participants through the trial.

*Participants Recruited and Included in the Analysis:* The study participants were recruited from August 25th, 2021, through February 24th, 2022. A total of 153 potential participants responded to the study advertisement. Of these, we could not reach 6 for phone screening despite multiple attempts; another 4 declined to pursue clinical screening; and 143 were screened through a structured telephone interview. After the phone screening, 60 potential participants were ineligible for clinical screening and 83 were eligible. Eleven eligible potential participants were unable to commit to the study, leaving us with 72 to clinically screen. Of those who were clinically screened, 19 did not meet the study eligibility criteria of risk for T2DM, while 53 met the eligibility. Thirteen of those who met the criteria for at risk for T2DM withdrew from the study before the initiation of the interventions, 1 was waitlisted, and we enrolled 39 participants in the study. The 39 participants were then randomized to 1 of 2 possible sequence permutations. The participants’ flow through the study is depicted in Fig. 1.

The study participants were predominantly female, 28 (71.8 %). The average age of the participants was 64.4 ± 8.2 years. The participants were obese with a mean BMI of 33.0 ± 6.0 kg/m^2^. Fifty-four percent of participants had a BMI ≥30 kg/m^2^ with Percent Body Fat ≥30. Most of the participants (61.5 %) were pre-diabetes. The participants’ average HbA1c at baseline was in the pre-diabetes range (5.8 ± 0.3 %) and they were at high risk for CVD with their average hs-CRP >3 mg/L. Demographic characteristics and baseline values of the study participants are presented in Table 1. Five participants were lost to follow-up: 1 participant was unable to resolve scheduling challenges; 3 participants stopped responding to follow-up calls and did not show up at scheduled visits; and 1 person dropped out due to health reasons.

**Table 1:** Demographic characteristic and baseline values in adults at risk for T2DM.

Variable	Values^a^(n = 39)
Gender	
Female, n (%)	28(71.8 %)
Male, n (%)	11(28.2 %)
Race	
Caucasian, n (%)	38(97.4 %)
African American, n (%)	1(2.6 %)
Age, Years	64.4 ± 8.2
Diabetes Risk Status	
Pre-diabetes, n (%)	24(61.5 %)
Metabolic Syndrome, n (%)	5(12.8 %)
Pre-diabetes and Metabolic Syndrome, n (%)	10(25.6 %)
Serum Glycemic Control	
Glycated Hemoglobin A1C, %	5.8 ± 0.3
Fasting Blood Glucose, mg/dL	106.8 ± 10.2
Insulin, mIU/L	12.8 ± 5.1
Homeostatic Model Assessment for Insulin Resistance	3.4 ± 1.5
Body Composition	
Weight, kg	90.0 ± 21.3
Body Mass Index, kg/m^2^	33.0 ± 6.0
Waist Circumference, cm	106.8 ± 15.6
Body Fat, %	38.8 ± 10.8
Body Water, %	42.7 ± 6.3
Visceral Fat Rating	13.7 ± 4.7
BMI ≥ 30 with Percent Body Fat≥30, n(%)	21(53.8 %)
Blood Pressure	
Systolic, mmHg	130.9 ± 14.4
Diastolic, mmHg	77.4 ± 8.8
Mean Arterial Blood Pressure, mmHg	95.2 ± 9.8
Inflammatory Marker	
High Sensitivity C-Reactive Protein, mg/L	3.5 ± 3.2
Endothelial Function	
Flow Mediated Dilatation, %	16.1 ± 9.0
Serum Lipid Profile	
Total cholesterol, mg/dL	196.3 ± 44.8
HDL cholesterol, mg/dL	58.0 ± 15.8
LDL cholesterol, mg/dL	113.3 ± 41.8
Triglycerides, mg/dL	131.0 ± 52.4
Total-/HDL cholesterol	3.4 ± 0.9
Pittsburgh Sleep Quality Index	5.4 ± 3.0
Liver Function	
Aspartate Transferase, U/L	29.0 ± 8.8
Alanine Aminotransferase, U/L	26.6 ± 14.9
Kidney Function	
Creatinine, mg/dL	0.9 ± 0.2

a Values are mean ± SD except otherwise stated, n = 39; T2DM, Type 2 diabetes mellitus.

*Efficacy Endpoint:* Compared with the placebo, the Fos Biomedical NTPS did not improve HbA1c scores (0.1 ± 0.2 vs. 0.1 ± 0.2; p = 0.5154) or HOMA-IR (0.01 ± 1.1 vs. −0.2 ± 1.4; p = 0.4519). Conversely, compared with the placebo, the Fos Biomedical NTPS significantly reduced endothelial function (−1.7 ± 12.1 vs. 3.9 ± 10.0; p = 0.0344). The Fos Biomedical NTPS compared with the placebo did not significantly (p > 0.05) improve body composition, blood pressure, lipid profile, hs-CRP, and sleep quality. Compared with the placebo, the Fos Biomedical NTPS did not adversely (p > 0.05) affect markers of liver and kidney function (see Table 2). Compared with the placebo, the study participants maintained a relatively stable physical activity level (p > 0.05) while using the Fos Biomedical NTPS. Likewise, the reported intake of calories, protein, carbohydrates, cholesterol, and fats did not differ significantly (p > 0.05) between Fos Biomedical NTPS compared with the placebo (see Table 3.). The compliance rate for using Fos Biomedical NTPS compared with the placebo was somewhat similar (81.0 % vs. 87.8 %; p = 0.3905).

**Table 2:** Change in outcome measures from baseline to 12 Weeks in adults at risk for T2DM with the use of Fos Biomedical patch compared with placebo.

Variable	Fos Biomedical Patch a	Placebo a	p-value (95 % CI)
Glycemic Control			
Glycated Hemoglobin A1C, %			
Baseline	5.8 ± 0.3	5.9 ± 0.3	
12-week	6.0 ± 0.3	5.9 ± 0.3	
Change	0.1±0.2^b^	0.1±0.2^b^	0.5154 (−0.1 to 0.1)
Homeostatic Model Assessment for Insulin Resistance			
Baseline	3.2 ± 1.6	3.1 ± 1.3	
12-week	3.2 ± 1.6	3.1 ± 1.5	
Change	−0.2 ± 1.4	0.01 ± 1.1	0.4519 (−0.9 to 1.1)
Body Composition			
Waist Circumference, cm			
Baseline	105.8 ± 14.6	105.6 ± 14.7	
12-week	103.8 ± 14.4	104.0 ± 15.0	
Change	−1.2±2.3^b^	−1.0±2.1^b^	0.6322 (−1.3 to 1.9)
Weight, kg			
Baseline	90.1 ± 22.2	88.3 ± 19.7	
12-week	90.1 ± 22.0	88.9 ± 19.8	
Change	−0.0 ± 1.5	0.5±1.6^b^	0.1546 (−1.2 to 1.3)
Body Mass Index, kg/m^2^			
Baseline	33.2 ± 6.4	32.6 ± 5.5	
12-week	33.5 ± 7.0	32.9 ± 5.7	
Change	0.3 ± 1.3	0.3±0.8^b^	0.9744 (−0.5 to 0.9)
Body Fat, %			
Baseline	40.2 ± 10.1	40.4 ± 9.5	
12-week	41.9 ± 8.7	41.3 ± 8.9	
Change	1.2 ± 6.7	1.0 ± 4.9	0.8766 (−2.5 to 5.0)
Body Water, %			
Baseline	42.2 ± 5.9	42.3 ± 5.7	
12-week	41.7 ± 5.7	41.8 ± 5.6	
Change	−0.1 ± 2.1	−0.5 ± 1.4	0.4049 (−0.5 to 1.5)
Visceral Fat Rating			
Baseline	14.0 ± 5.6	14.3 ± 4.9	
12-week	14.8 ± 5.3	15.1 ± 6.1	
Change	0.6 ± 2.6	0.8±2.0^b^	0.8065 (−1.2 to 2.0)
Blood Pressure			
Systolic, mmHg			
Baseline	128.8 ± 14.9	128.2 ± 15.3	
12-week	130.0 ± 13.1	129.9 ± 19.7	
Change	1.3 ± 12.5	1.6 ± 15.8	0.9055 (−7.1 to 12.3)
Diastolic, mmHg			
Baseline	76.9 ± 8.6	76.1 ± 8.3	
12-week	77.4 ± 7.1	76.2 ± 8.7	
Change	0.5 ± 6.8	0.2 ± 7.0	0.8481 (−2.9 to 5.9)
Mean Arterial Blood Pressure, mmHg			
Baseline	94.2 ± 10.0	93.4 ± 9.9	
12-week	94.9 ± 7.7	94.1 ± 10.6	
Change	0.7 ± 7.5	0.7 ± 8.5	0.9689 (−3.7 to 6.9)
Inflammatory Marker			
High Sensitivity C-Reactive Protein, mg/L			
Baseline	3.4 ± 3.1	3.1 ± 2.7	
12-week	3.3 ± 2.6	2.9 ± 2.5	
Change	−0.2 ± 3.6	0.2 ± 2.9	0.6610 (−2.0 to 2.8)
Endothelial Function			
Flow Mediated Dilatation, %			
Baseline	18.0 ± 10.7	14.4 ± 8.4	
12-week	16.2 ± 7.7	18.2 ± 10.1	
Change	−1.7 ± 12.1	3.9±10.0^b^	0.0344 (−10.9 to 9.5)
Serum Lipid Profile			
Total cholesterol, mg/dL			
Baseline	193.3 ± 40.3	195.5 ± 43.4	
12-week	194.5 ± 39.8	204.0 ± 39.7	
Change	2.7 ± 25.2	7.0 ± 25.8	0.4755 (−16.4 to 21.9)
HDL cholesterol, mg/dL			
Baseline	59.1 ± 15.0	58.5 ± 15.0	
12-week	55.4 ± 14.9	55.8 ± 15.3	
Change	−3.3 ± 13.1	−3.1±6.7^b^	0.9354 (−5.1 to 8.9)
LDL cholesterol, mg/dL			
Baseline	110.0 ± 38.4	111.9 ± 40.4	
12-week	111.4 ± 36.4	120.9 ± 37.2	
Change	2.5 ± 22.3	7.6 ± 23.7	0.3545 (−16.0 to 19.7)
Triglycerides, mg/dL			
Baseline	128.3 ± 55.0	124.9 ± 50.2	
12-week	139.1 ± 78.7	136.4 ± 48.8	
Change	10.1 ± 41.5	12.7 ± 38.4	0.7865 (−21.5 to 34.3)
Total-/HDL cholesterol, %			
Baseline	3.3 ± 0.8	3.4 ± 1.1	
12-week	3.8 ± 1.2	3.9 ± 1.2	
Change	0.5±0.9^b^	0.4±0.8^b^	0.8546 (−0.4 to 0.7)
Pittsburgh Sleep Quality Index			
Baseline	5.1 ± 3.0	5.6 ± 3.1	
12-week	5.1 ± 3.5	4.9 ± 2.9	
Change	−0.03 ± 2.9	−0.4 ± 2.0	0.5258 (−0.8 to 2.2)
Sleep Disturbance			
Baseline	1.3 ± 0.5	1.4 ± 0.7	
12-week	1.3 ± 0.7	1.2 ± 0.5	
Change	0.03 ± 0.5	−0.2 ± 0.6	0.0977 (−0.05 to 0.5)
Liver Function			
Aspartate Transferase, U/L			
Baseline	30.8 ± 9.2	28.9 ± 7.2	
12-week	29.5 ± 8.9	30.0 ± 6.4	
Change	−1.2 ± 5.6	1.1 ± 5.5	0.0830 (−4.9 to 4.7)
Alanine Aminotransferase, U/L			
Baseline	29.2 ± 18.7	26.6 ± 13.6	
12-week	30.5 ± 15.7	29.6 ± 13.2	
Change	1.6 ± 9.8	3.0±7.5^b^	0.4868 (−5.5 to 7.4)
Kidney Function			
Creatinine, mg/dL			
Baseline	0.9 ± 0.2	0.9 ± 0.2	
12-week	0.8 ± 0.2	0.8 ± 0.2	
Change	−0.03 ± 0.1	−0.03±0.1^b^	0.7706 (−0.03 to 0.08)

^3^ Confidence Interval; T2DM, Type 2 diabetes mellitus.

a Values are mean ± SD, n = 39.

b significant (p < 0.05) change from baseline.

**Table 3:** Change in physical activity and dietary pattern from baseline to 12 Weeks in adults at risk for T2DM while using Fos Biomedical patch compared with placebo.

Variable	Fos Biomedical Patch a	Placebo a	p-value (95 % CI)
Physical Activity			
Total Weekly Energy Expenditure (kcal/kg/wk)			
Baseline	75.5 ± 60.3	61.6 ± 45.1	
12-week	70.7 ± 55.3	73.6 ± 70.7	
Change	−5.4 ± 44.1	12.0 ± 66.1	0.1786 (−43.0 to 48.6)
Dietary Pattern			
Daily Calorie Intake (kcal)			
Baseline	1702.2 ± 491.7	1720.6 ± 452.0	
12-week	1659.3 ± 524.0	1652.8 ± 358.3	
Change	−31.4 ± 616.2	−93.2 ± 428.	0.6449 (−202.0 to 448.9)
% Calories from Protein			
Baseline	18.1 ± 4.2	17.4 ± 4.6	
12-week	18.8 ± 4.1	18.5 ± 5.9	
Change	0.6 ± 3.8	1.2 ± 4.8	0.6388 (−2.7 to 3.7)
% Calorie from Total Fat			
Baseline	36.1 ± 6.3	37.7 ± 5.9	
12-week	37.1 ± 6.3	38.1 ± 7.4	
Change	1.4 ± 6.7	−0.1 ± 6.7	0.3746 (−1.8 to 5.7)
Daily Dietary Cholesterol Intake (mg)			
Baseline	293.7 ± 157.6	293.2 ± 171.2	
12-week	296.6 ± 118.2	313.5 ± 159.8	
Change	7.2 ± 164.1	9.5 ± 174.6	0.9572 (−87.1 to 144.3)
%Calories from Saturated Fats			
Baseline	11.7 ± 3.6	12.1 ± 2.8	
12-week	12.1 ± 3.0	12.5 ± 3.7	
Change	0.6 ± 3.3	0.2 ± 3.8	0.6221 (−1.3 to 3.0)
%Calories from Monounsaturated Fats			
Baseline	12.7 ± 2.4	13.2 ± 3.5	
12-week	13.1 ± 2.9	13.6 ± 3.6	
Change	0.3 ± 3.1	0.2 ± 3.4	0.9086 (−1.5 to 2.8)
%Calories from Polyunsaturated Fats			
Baseline	8.6 ± 2.4	8.9 ± 2.5	
12-week	8.8 ± 2.2	8.6 ± 2.5	
Change	0.4 ± 3.1	−0.4 ± 2.8	0.3186 (−0.7 to 2.5)
%Calories from Carbohydrates			
Baseline	45.5 ± 8.5	43.8 ± 8.7	
12-week	43.9 ± 7.8	42.0 ± 9.3	
Change	−1.9 ± 7.8	−1.5 ± 8.2	0.8112 (−4.5 to 6.8)

^3^ Confidence Interval; T2DM, Type 2 diabetes mellitus.

^2^ significant (p < 0.05) change from baseline.

a Values are mean ± SD, n = 39.

*Adverse Events:* Eight adverse events were reported during the study interventions. A participant reported redness in the abdomen where the patches were applied in both treatment phases; another participant reported rash in the lower abdomen 2 days after wearing the patches; another participant reported petechiae rash where the patches were applied; a participant reported developing rashes from the patches about 5 weeks into the intervention; another reported developing a minor rash 2 weeks into the intervention, which quickly resolved; another reported minor skin irritation from the patches; and a participant complained of abdominal tenderness that developed during the study intervention.

## 5 Discussion

Glycemic control, body composition, blood pressure, lipid profile, inflammation, and sleep quality did not significantly improve after using Fos Biomedical NTPS. Daily use of the Fos Biomedical NTPS for 12 weeks among those at risk for T2DM significantly reduced endothelial function. Dietary and physical activity patterns were relatively stable throughout the study. Likewise, liver and kidney function were unaffected.

We did not observe any significant improvement in glycemic control, body composition, blood pressure, lipid profile, and inflammatory biomarkers following the use of Fos Biomedical NTPS in our participants at risk for T2DM. However, previous studies [16,17,[30], [31], [32]], mostly conducted on animal models except one conducted on humans, have shown an association between PBM therapy and improvement of these measures. Specifically, Gong et al. [16] demonstrated improved glucose metabolism and insulin sensitivity with PBM therapy, by activating CCO-protein kinase B in the muscles of T2DM mice. Another study by Gong et al. [30], demonstrated that PBM therapy reduced free fatty acid generation and release in white adipose tissues to improve glycemic control in diabetes mice fed with a high-fat diet. Furthermore, low-power laser irradiation suppresses excessive lipolysis in insulin-resistant adiposity by activating the tyrosine kinases-1(Dok1)/ERK/PPARγ pathway [31]. Additionally, Yoshimura et al. [17] demonstrated that 6 sessions of PBM therapy, compared with control, significantly reduced abdominal inflammatory infiltrate in diet-induced obese hyperglycemic mice. Also, irradiation with UVA phototherapy has been shown to transiently reduce blood pressure in patients with mild hypertension [32]. Unfortunately, most previous studies examining the effects of PBM on these measures were conducted on animal models, while ours focused on humans. Therefore, there are significant variations in ethical considerations and physiological responses. These inherent biological differences could limit the applicability of the findings of previous studies to our human study. Additionally, in contrast to the findings in these previous reports, we did not observe any meaningful improvements in these measures in our study, possibly due to differences in study designs; inadequate sample size; insufficient duration of study intervention; inappropriate utilization of the patch system by the study participants; and/or deactivation of the Fos Biomedical NTPSs through exposure to extreme heat or electromagnetic fields during transportation, resulting in suboptimal levels of light in the infrared and visible bands that were generated.

Phototherapy has been associated with improved cardiovascular health in previous studies with small samples and methodological flaws [15,[33], [34], [35]]. Specifically, in a retrospective cohort study by Bae et al. [33], long-term narrowband ultraviolet B phototherapy was associated with a lower risk of cardiovascular and cerebrovascular events among patients with vitiligo who were treated with the phototherapy compared with those who were not. Also, phototherapy compared with placebo has been shown to improve symptoms of microvascular complications in diabetes patients [15]. Likewise, Plass et al. [34] demonstrated that light was associated with dilatation of coronary arteries. Moreover, Plass et al. [35] demonstrated that low-level laser irradiation was associated with endothelium-dependent vasodilatation. In effect, near-infrared PBM has been shown to enhance NO bioavailability through phosphorylation of NO synthase in endothelial cells [36]. Similarly, low-intensity light has been shown to stimulate nitrite-dependent NO synthesis that is catalyzed by CCO [37]; which helps to explain the increase in the bioavailability of NO that is experienced by tissues exposed to light. Contrary to these previous reports and our study hypothesis, we observed a reduction in endothelial function (i.e., endothelium-dependent vasodilatation) after daily application of the Fos Biomedical NTPS for a 12-week period compared with placebo in participants at risk for T2DM. The surprising finding observed in endothelial function, could possibly have been due to a placebo effect combined with inadequate PBM generated by the Fos Biomedical NTPS or seasonal variation.

We did not observe any meaningful improvement in sleep quality after our participants used the Fos Biomedical NTPS daily for a 12-week period compared with placebo. In a meta-analysis by Van Maanen et al. [38] light therapy was associated with improvement in circadian rhythms and insomnia. We did not observe any meaningful improvement, possibly due to deactivation of the patch system through exposure to electromagnetic fields or extremely high temperatures, a small sample size, and/or the placebo effect.

## 6 Limitations

It is important to consider some potential limitations of the study as we interpret these data. First, the sample size was small, with just 39 participants enrolled, which may have precluded us from seeing statistically significant differences. The study's crossover design reduced our data variation and to some degree improved the study's statistical power. Second, our study participants were predominantly Caucasians and middle-to older-aged women; therefore, this limits our ability to extrapolate our findings to other racial and ethnic groups. Third, the study participants were not monitored daily, and their patches were not administered under supervision. The veracity of the adherence to the utilization of the intervention patches relied on the feedback provided by the participants and their adherence log. However, this could also be viewed as a strength of the study because it provided a more realistic scenario and theoretically improved external validity. Fourth, this study relied on self-report by the participants regarding their dietary intake, physical activity level, and sleep quality, which can introduce measurement and recall biases by under- or over-estimating. However, the data were captured using reliable, validated instruments that provided guidance to the participants, which reduced the chance of under- or over-estimation. Fifth, another source of limitation may stem from the inherent day-to-day variability of the participants' diets and physical activity levels. Nonetheless, averaging the self-reported 3-day (i.e., 2 weekdays and 1 weekend day) intake of foods and beverages and using the 7-day physical activity level by each participant to some extent adjusted for the day-to-day variation to arrive at scores closer to the participants' usual dietary intake and physical activity habits. Sixth, we did not see improvement in cardiometabolic health and sleep quality, possibly because of suboptimal levels of PBM generated by the Fos Biomedical NTPS. The patch systems may have been deactivated during transportation through exposure to electromagnetic fields and/or extremely high temperatures.

## 7 Conclusion

Despite the aforementioned limitations, our data suggests that short-term daily application of the Fos Biomedical NTPS did not improve cardio-metabolic risk factors and sleep quality in participants at risk for T2DM. To better understand the effects of the Fos Biomedical NTPS on cardio-metabolic health and sleep quality among those at risk for T2DM, a rigorous procedure is warranted to ascertain that the Fos Biomedical NTPS are protected from exposure to electromagnetic fields and extremely high temperatures during transportation, storage, and application to safeguard adequate PBM generation. In addition, large, well-designed, randomized controlled trials on humans are necessary to clarify the benefits of PBM for cardio-metabolic health and sleep quality.

## CRediT authorship contribution statement

**Valentine Y. Njike:** Writing – original draft, Supervision, Methodology, Investigation, Formal analysis, Conceptualization. **Rockiy G. Ayettey:** Project administration, Data curation. **Judith A. Treu:** Writing – review & editing. **Beth Patton Comerford:** Writing – review & editing, Supervision, Project administration. **Maureen Onuigbo:** Writing – review & editing.

## Disclosure Statement

The authors have nothing to disclose.

## financial support

This study was conducted with funding from Fos Biomedical and the 10.13039/100000030Centers for Disease Control and Prevention. The funder of the study had no role in study design, data collection, data analysis, data interpretation, or writing of the manuscript.

## Declaration of competing interest

The authors declare the following financial interests/personal relationships which may be considered as potential competing interests: Valentine Y. Njike, MD, MPH reports financial support was provided by Fos Biomedical. Valentine Y. Njike, MD, MPH reports a relationship with 10.13039/100000030Centers for Disease Control and Prevention that includes funding grants. If there are other authors, they declare that they have no known competing financial interests or personal relationships that could have appeared to influence the work reported in this paper.

## Data Availability

Data will be made available on request.
